# Identifying Affecting Factors on Acceptance with CPAP on the First Night of PAP Titration in Sleep Clinic on Patients with Obstructive Sleep Apnea 

**DOI:** 10.22038/IJORL.2023.68296.3325

**Published:** 2023-11

**Authors:** Reihaneh Heidari, Ayeh Shamsadini, Arezu Najafi, Reza Erfanian

**Affiliations:** 1 *Otorhinolaryngology Research Center, Imam Khomeini Hospital Complex, Tehran University of Medical Sciences, Tehran, Iran. *; 2 *Occupational Sleep Research Center, Baharloo Hospital, Tehran University of Medical Sciences, Tehran, Iran. *; 3 *Otorhinolaryngology Research Center, Tehran University of Medical Sciences, Tehran, Iran. *

**Keywords:** Acceptance, CPAP, OSA

## Abstract

**Introduction:**

This research examined the causes of low acceptance with Continuous Positive Airway Pressure Continuous Positive Airway Pressure (CPAP) especially anatomical causes and if eliminating them would result in increasing its adherence.

**Materials and Methods:**

This cross sectional study was performed on patients with moderate to severe Obstructive Sleep Apnea Obstructive Sleep Apnea (OSA) undergoing PAP titration in the sleep clinic. CPAP acceptance was evaluated by visual analog scale (VAS) about mask and sleep satisfaction and the possibility of using CPAP in the future, mask complications, physical examination of the upper airway and polysomnographic (PSG) results before and after titration.

**Results:**

participants were divided into three groups of non-acceptant, semi-acceptant and acceptant with CPAP based on the satisfaction of the mask and sleep. There were no significant differences between groups based on age, gender, education, BMI and polysomnographic variables. With a study of mask complication, there were significant differences among groups for dry mouth, mask leakage and cold air. (p<0.05) The severity of septal deviation, high arch palate, mallampati, retrognathia and maxillary hypoplasia in the acceptant group was less than the other two groups, but it was not statistically significant.

**Conclusions:**

Satisfaction with the sleep and the mask on the first night of titration will significantly increase the likelihood of using CPAP in the future. A number of the pathological physical examinations were lower in the acceptant group than two other groups, but were not significant.

## Introduction

Obstructive sleep apnea (OSA) is regarded as a common sleep disorder which is characterized by upper airway collapse which leads to a decrease or cessation of airﬂow during sleep (1). Both upper airway narrowing and increased airway resistance may play an important role in pathogenesis of OSA which results in symptoms of loud snoring, apnea, and systemic complications in the case of not being treated properly (2). 

So, researchers have proposed different therapeutic options such as medical or surgical treatment in order to improve upper airway narrowing and also decrease airway resistance in OSA patients. CPAP is regarded as the standard treatment for patients with moderate-to-severe OSA (3). 

CPAP is a device that transfers air via a nasal or oronasal mask at a fixed pressure that remains constant throughout the respiratory cycle. The proposed mechanism of CPAP therapy is that it acts as a pneumatic splint which maintains the patency of the upper airway in a dose-dependent fashion (4). The clinical benefits of CPAP treatment such as the improvement of subjective symptoms and life-threatening conditions have been indicated by many researchers. (5-10). In spite of its recognized beneﬁts, technological improvements and efforts to enhance usability, CPAP adherence is generally poor, and its use is often bothersome (11-13). 

Multiple factors which have an impact on adherence to CPAP therapy include subjective sleep-related symptoms (12), OSA severity (13), knowledge of CPAP’s effects as well as side effects and discomfort (14). 

Additionally, anatomic structure would play a role in CPAP adherence. Also, narrow upper airway anatomy might be related to therapeutic positive airway pressure (PAP) and discomfort. Plus, it has been reported that CPAP compliance could be improved with optimal treatment of narrowed anatomic structures in a medical or surgical way (15,16). 

Therefore, further studies are required to assess the upper airway anatomy of OSA patients and to measure various parameters in patients who receive CPAP for OSA treatment. The reason is to ultimately improve compliance with CPAP therapy. In contrast, few studies have reported the physical differences between adherent and non-adherent CPAP patients. On the other hand, research has indicated that short-term acceptance of CPAP can predict long-term compliance (17,18). Given that most of the studies were on long-term CPAP compliance, we decided to do this study and review the short-term acceptance (first night of titration) and that by knowing the factors affecting the poor acceptance of CPAP and eliminating them, especially anatomical causes with surgery can increase its compliance.

## Materials and Methods

This cross-sectional study was performed on patients with moderate to severe obstructive sleep syndrome undergoing PAP titration in the sleep clinic. Inclusion criteria was considered as performing titration in the clinic, Apnea Hypopnea Index (AHI) moderate to severe and also age between 20 and 70 years. 

Likewise, exclusion criteria were Previous use of CPAP, patient dissatisfaction, having proven psychiatric disorders and performing split night Polysomnography (PSG). The level of CPAP acceptance of patients after obtaining consent was evaluated in the morning after titration by several methods including: 

1- Visual Analog Scale (VAS) and a score given by the patient from 0 to 10 about satisfaction with the mask, satisfaction with the sleep and the possibility of using CPAP in the future. 

 2-Self-Reported Questionnaire (about compli- cations of the mask)

3- PSG results before and after titration 

4-The results of the physical examination of the patients’ upper airway. 

A complete facial and upper airway examination was performed by an ENT and sleep specialist. The anatomical examination included the following: mallampati, septal deviation, tonsillar hypertrophy, turbinate hypertrophy, posterior airway space, high arch palate, retrognathia ،maxillary hypoplasia, nasal polyp, external nasal deformity and tongue hypertrophy. 

After recording the information, participants were divided into three groups: non-acceptant (first group), semi-acceptant (second group) and acceptant (third group). 

Each of the variables in each group was examined and compared between groups. The recorded data in the forms was entered into the SPSS software. Mean and standard deviation were used to describe quantitative variables and also frequency and percentage were used to describe qualitative variables. To compare the means, t-test was used in two groups and one-way analysis of variance was used in three groups. Chi-square test was used to compare qualitative variables. Logistic regression was used to investigate the relationship between factors which related to patient admission. 

Due to the importance of accepting the first night of mask use, patients' satisfaction with the mask and sleep was questioned. By converting VAS scores from 0 to 10 to percentage values, a score of 0 was considered as absolute dissatisfaction and a score of 100 was considered as excellent satisfaction. In terms of comparing sleep satisfaction and mask satisfaction, the median scores showed that in most participants, sleep satisfaction was slightly higher than mask satisfaction.

 The results show that about one third of the surveyed people have given an average satisfaction score of less than 45% and about one third of the satisfaction score of 70% or more to their sleep and mask. 

Accordingly, individuals are divided into three groups in terms of satisfaction: non-acceptant (less than 45% of participants), semi-acceptant (45% to less than 70%), and acceptant (70% or more) to this method. Classification and other analyzes are based on this category. 

*Ethical considerations*

Written consent was obtained from all participants and the personal information of all patients remained confidential. The information was analyzed in groups without name and surname and the given code of ethics wasIR. TUMS. FNM. REC.1399.053

## Results

  Out of 78 participants, 25 were in the first group (non-acceptant), 27 were in the second (semi-acceptant) and 26 were in the third group (acceptant). Of 78 subjects, 60(76.9%) were male and 18(23.1%) female. 

The mean age was 46.17+-10.62 and the minimum and maximum age values ​​ranged from 20 to 67. The mean Body Mass Index Body Mass Index (BMI) score of patients was 30.01± 6.89. The mean Apnea and Hypopnea Index (AHI) and Epworth Sleepiness Scale Excessive Sleepiness Scale (ESS) was 59.82+-26.1 and 11.82+-6.03 respectively. 

There were no statistically significant differences between the three groups in terms of age, gender, level of education, BMI and ESS. In terms of education level and acceptance of the mask, the study also showed that people with a lower level of education than the diploma, accepted the mask a little better than other people with higher education. However, this difference between the groups was not statistically significant. (p>0.8) (Table1).

**Table 1:** clinical characteristic of subjects

	**Total**	**Non- acceptant ** ** (n=25)**	**Semi-acceptant (n=27)**	**Acceptant** ** (n=26)**	**P.value**
Age	46.17+-10.62	18.04+-10.10	44.70+-9.30	44.85+-12.41	0.547
Gender	M 60(76.9%) F 18(23.1%)	M 20(80%) F 5(20%)	M 21(77.8%) F 6(22.2%)	M 19(76.9%) F 7(26.9%)	0.372
BMI	30+-6.89	29.91+-7.14	28.82+-5.34	31.33+-8.03	0.421
ESS	11.82+-6.03	13.24+-6.24	10.07+-6.13	12.27+-5.48	0.151
Education	U/D 37 (47.4%) O/D 41 (52.6%)	14(37.8%) 11(44%)	8(21.6%) 19 (59.4%)	15(40.5%) 11 (52.4%)	0.514
					

*Polysomnographic variables*

By studying the polysomnographic variables (N1, N2, N3, REM, sleep latency and sleep efficiency), no significant difference was observed between the three groups (p>0.05). REM.P2(REM in second night of test, PAP titration) was greater in the third group, but was not significant. In the study of the average score of Efficiency among individuals in terms of mask acceptance, it was observed that only in the non-acceptant group, the average of this index in the second night of test (with mask) was slightly reduced compared to the first night of test (PSG) without mask which was statistically significant. (p<0.05) (Table 2).

**Table 2:** Comparison of the average score of Efficiency in the two sessions before and after using the mask in non-acceptant group

**Non acceptant group**	**Sleep. Efficiency. p1**	**Sleep. Efficiency. p2**
Percent	79.51%	70.78%
Standard deviation	9.61	18.9

*Mask complications*

The simple frequency of different side effects of the mask in all patients shows that 160 cases were reported among 78 patients. The three most common complications among patients were dryness (58.9%), mask leakage (37.17%) and dermatitis (26.9%) (Table 3).

**Table 3:** Simple and relative frequency of occurrence of side effects of mask use

**Mask Complications**	**Number (Percent)**
Dermatitis	21 (26.9%)
Rhinitis	3 (3.8%)
Dryness	46 (58.9%)
Claustrophobia	3 (3.8%)
Aerophagia	18(23.07%)
Barotrauma	9 (11.53%)
Cold Air	5 (6.41%)
Choking Sensation	18 (23.07%)
Leakage	29(37.17%)

 With a separate study of each complication, there was a significant difference between the three groups for dry mouth, mask leakage and cold air. (p<0.05)

Among the three groups of acceptant, semi-acceptant and non-acceptant, a significant difference was observed in terms of incidence of at least one of the ten complications (mask leakage, dermatitis, rhinitis, dry mouth, claustrophobia, aerophobia, barotrauma , chest discomfort , cold air and choking sensation) (p<0.05) At least one complication occurred in 19 cases (71.3%) of those who accepted the mask, but in other groups that were semi-acceptant or non-acceptant, it was 96.3% and 88% of the cases respectively (Table 4).

**Table 4:** Incidence of at least one side effect of the mask and comparison of its frequency inof mask acceptance

**Group**	**Positive**	**Percent**
Acceptant	19	71.3%
Semi- acceptant	26	96.3%
Non-acceptant	22	88%
Total	67	78.2%

*Physical examination results*

Out of 78 participants, only 4 had no pathological signs on physical examination. The highest prevalence of pathological physical examination was septal deviation, reduction of PAS (posterior airway space, space between uvula and posterior pharyngeal wall) and greater mallampati score, respectively. In the physical examination, the rate of septal deviation, high arch palate, mallampati, retrognathia and maxillary hypoplasia in the third group was considered less than the other two groups, but it was not statistically significant. (Table 5) Septal deviation as the most common symptom in all patients with a prevalence of 35 cases out of 78 patients (44.9%) was slightly different from other cases.

 Additional findings: the study with chi-square test showed that the only case in which the two sexes differed was septal deviation, in which 31 out of 60 (51.7%) men had this problem and among women only 4 out of 18 (22.2%) were positive and this difference was significant. (p<0.05). 

Among other symptoms, external nasal deformity was seen in only one female patient and was not present in men. Also, no nasal valve stenosis was and no nasal polyps were observed in any of the 78 patients. (Table 5) To clarify the relationship between mask acceptance and physical examination patients with at least one pathological examination were compared. Mainly those who did not accept the mask or were semi-acceptant had at least one pathological problem in the physical examination, and among those who accepted the mask, the percentage of pathological complication was slightly lower than that of the semi-receptive or non-acceptant. But there wasn’t a significant difference between the groups. 

Examining the mean score given by patients with VAS as a possibility of using the mask in the future, it was found that people who accepted the mask scored higher than those who were semi-acceptant or non-acceptant, and their mean scores was statistically significant. (p<0.05) (Table 6).

**Table 5:** Comparison of simple and relative frequency of pathological symptoms in physical examinations according to mask acceptance

**Pathologic Physical exam**	**Total**	**Non-acceptant (n=25)**	**Semi-acceptant (n=27)**	**Acceptant (n=26)**	**p.value**
Mallampati	26(33.3%)	11(44%)	10(37%)	5(19.2%)	0.152
Septal deviation	35(44.9%)	14(56%)	11(40.7%)	10(38.5%)	0.393
Tonsillar hypertrophy	8(10.3%)	2(8%)	2(7.4%)	4(15.4%)	0.571
Turbinate hypertrophy	27(34.61%)	8(32%)	8(29.6%)	9(34.6%)	0.927
Posterior airway space reduction	33(42.3%)	10(40%)	13(48.1%)	10(38.5%)	0.745
High arch palate	20(25.6%)	9(36%)	8(29.6%)	3(11.5%)	0.114
Retrognathia	14(17.9%)	5(20%)	6(22.2%)	3(11.5%)	0.568
Tongue hypertrophy	21(26.9%)	6(24%)	8(29.6%)	7(26.9%)	0.901
Maxillary hypoplasia	6(7.69%)	4(16%)	2(7.4%)	0	0.306
External nasal deformity	1(1.28%)	1(4%)	0	0	
At least having one symptom	74(94.78%)	25(100%)	26(96.3%)	22(84.6%)	>0.06

**Table 6:** Comparison of the average VAS score for the likelihood of using the mask in the future in terms of mask acceptance

**Group**	**Number**	**Mean future VAS**	**Std.deviation**
Non-acceptant	25	3.12	2.651
Semi-acceptant	27	4.81	2.936
Acceptant	26	7.27	2.290
Total	78	5.09	3.113

## Discussion

 The current research demonstrates that mask and sleep satisfaction are correlated with CPAP acceptance in the first night of PAP titration as well as using CPAP in the future. Therefore, the study indicates that anatomic factors like septal deviation, high arch palate, retrognathia, mallampati and maxillary hypoplasia can provide special information that correlate with CPAP acceptance. 

 In the study by Pona park higher grades of septal deviation and hypertrophy of the inferior turbinate were observed more in the CPAP non-adherent group. In addition, CPAP non-adherent subjects showed considerably bigger tonsils and higher grade palatal position comparing with the CPAP adherent subjects, and Subjective discomfort including inconvenience, mouth dryness, and chest discomfort were the main problems for OSA subjects who did not comply with CPAP therapy (2). Our study showed that most common side effects of the mask were dry mouth, mask leakage and dermatitis. No statistically significant differences were observed in sleep parameters between CPAP-adherent patients and CPAP non-adherent subjects in Pona park study and our study also. Chea hooi, conducted a research on 135 patients with moderate to severe OSA. The findings showed that OSA severity (AHI) and symptomatic improvement after CPAP were associated with better adherence. Meanwhile, Presence of machine related side effects was associated with lower adherence. Inconvenience, cost and poor disease perception were reported as major factors of CPAP non-adherent subjects (19). In our study, side effects of mask were correlated with CPAP acceptance, too. In 2008, Ranju et al carried out a research on 11 patients with severe OSA with poor CPAP compliance (1 hour and 58 minutes per night) for oral, nasal, or both surgeries.  After surgery, AHI decreased from 79 to 30 and mean CPAP compliance improved (2 hours and 46 minutes per night) (20). In 2007, Gosh et al. Examined 407 patients with varying degrees of OSS who were referred for CPAP. 82% of them accepted CPAP and 18% had no compliance. There were no significant differences between the two groups (21). In another study in 2002, Sin DD et al followed 296 patients with moderate to severe OSS at CPAP treatment at 2 weeks, 4 weeks, 3 months, and 6 months and found three variables; female gender, aging, and decreased ESS, were associated with increased CPAP compliance (22). The advantage of our study over previous studies was the study on the first night as well as the comparison of acceptance between the three groups while other studies had comparisons between the two groups.

## Conclusion

Satisfaction with sleep and mask on the first night of titration will significantly increase the likelihood of using CPAP in the future. Several of the pathological physical examinations were lower in the acceptant group than the semi-acceptant and non-acceptant second group. However, no statistically significant difference was observed, this can be due to the small sample size and patient desire. 

The limitation of this study was the small sample size that was less than calculated due to the increase in the price of CPAP. The advantage of this study over previous studies was the study on the first night as well as the comparison of acceptance between the three groups while other studies had comparisons between the two groups.

*Recommendation*

 Eliminating the anatomical defects of the upper airway before PAP titration to improve CPAP adherence. Performing further studies by comparison before and after upper airway surgery.
